# The Effect of Differentiation of Human Dental Pulp Stem Cells to Glial Cells on the Sensory Nerves of the Dental Pulp

**DOI:** 10.1155/2024/3746794

**Published:** 2024-04-05

**Authors:** Yan Liu, Yuxi Zou, Xinxin Liu, Yushan Wang, Jing Sun, Changyong Yuan, Penglai Wang

**Affiliations:** ^1^School of Stomatology, Xuzhou Medical University, Xuzhou, Jiangsu, China; ^2^Affiliated Stomatological Hospital of Xuzhou Medical University, Xuzhou, Jiangsu, China; ^3^School and Hospital of Stomatology, Cheeloo College of Medicine, Shandong University, Jinan, Shandong, China

## Abstract

Regeneration of sensory nerves is challenging in dental pulp regeneration. Schwann cells (SCs) are essential glial cells conducive to regenerating sensory nerve, but their source is scarce. The aim of the protocol was to investigate the regenerative potential of Schwann-like cells derived from dental pulp stem cells (SC-DPSCs) for sensory nerve regrowth. SC-DPSCs were generated from dental pulp stem cells using a three-step protocol. The expression of key markers, including myelin basic protein, S-100, and p75 neurotrophin receptor, was analyzed. Primary trigeminal neurons were cultured, and the expression of neurofilament 200, *β*-tubulin III, and microtubule-associated protein 2 was assessed. Simultaneous culture experiments were conducted to evaluate trigeminal neuron growth in the presence of SC-DPSCs. In addition, mRNA sequencing was performed to identify key genes involved in the differentiation process, highlighting prostaglandin-endoperoxide synthase 2 (PTGS2) as a potential candidate. The results demonstrated that SC-DPSCs expressed characteristic SCs markers and facilitated axonal growth in rat trigeminal nerves. Differentiated SC-DPSCs secreted elevated levels of nerve growth factors, including brain-derived neurotrophic factor and neurotrophin-3, promoting the growth of trigeminal nerve axons. These findings suggest the regenerative potential of SC-DPSCs in dentin–dental pulp complex; PTGS2 is considered a crucial gene in this differentiation process.

## 1. Introduction

Carious lesions, traumatic injuries, and developmental anomalies are major causes of pulp necrosis and apical periodontitis in immature permanent teeth [1], which eventually deter development of the root. This process makes the affected tooth vulnerable to external insults and loading, potentially leading to root fracture or tooth loss [2]. Apexification, apexogenesis, and pulp regeneration (revascularization) are three commonly performed clinical procedures [3]; however, regenerative endodontic treatment is superior in terms of pulp revitalization, root elongation, and wall thickening [4]. Two regenerative strategies are used: cellular regenerative and cell-free regenerative endodontic treatment based on exogenous stem cell transplantation and homing, respectively [5].

Sympathetic and sensory nerves innervate the pulp tissue and play important repair functions in other tissues, for example, sensory nerves innervating hair follicles regulate the response of hair follicle stem cells during injury repair [6]. Sympathetic innervation regulates the output of hematopoietic stem cells from the bone marrow [7]. Jacobsen and Heyeraas [8] demonstrated that sensory nerve fibers play an important role in promoting dentine formation and reduced sensory nerve supply slowed dentine formation. Neuropeptides such as CGRP and substance P released from sensory nerve endings can initiate healing responses associated with tissue injury. In addition, damage to pulp tissue leads to increased synthesis of nerve growth factor by pulp cells in the vicinity of adult dentin cells, followed by the appearance of CGRP and substance p immunoreactive nerve fibers [9], suggesting the functional importance of sensory during the pulp injury process. Zhao et al. [10] demonstrated that mesenchymal stem cell (MSC) favoring dentin regeneration originates from periarterial cells and is regulated by Shh secreted from the neurovascular bundle. Current research on sensory nerve regeneration has focused on the potential for MSC differentiation into central neural stem or neuronal cells [11]; however, the dental pulp sensory nerves originate from peripheral nerves, namely the trigeminal nerve maxillary and mandibular branches [12]. Schwann cells (SCs) are critical for nerve regeneration, including degenerated axon, myelin debris, and inhibitory factor elimination at the distal end, and provide vital topographical cues and specific proteins to guide axon elongation in bridging the interstump gap [13, 14]. Nevertheless, using primary SCs in cell-based regeneration has limitations, including slow proliferation and donor nerve tissue morbidity.

Human dental pulp stem cells (hDPSCs) have low immunogenicity and can generate pulp–dentin complexes; they demonstrate a strong neurogenic capacity and constitutively express various neural markers because of their neural crest origin [15]. hDPSCs have been used for repairing central nervous injuries and neurodegeneration and can differentiate into SCs, express SC markers, and secrete more neurotrophic factors compared with undifferentiated hDPSCs, thereby providing neuroprotection and nutritional support for axon regeneration [16]. Paracrine secretion and differentiation into glial cells are the central mechanisms through which hDPSCs exert therapeutic effects by promoting axonal outgrowth [17].

We aimed to differentiate hDPSCs into SCs and explore their capacity for axonal regeneration in trigeminal nerves. The differentially expressed genes (DEGs) were screened by transcriptome sequencing using a clustering heat map, gene ontology (GO), and Kyoto Encyclopedia of Genes and Genomes (KEGG) pathway analyses. The key differential genes were further screened by association networks, such as protein–protein interactions (PPIs) based on the Search Tool for the Retrieval of Interacting Genes/Proteins database and free energy analysis by relapse for mRNAs.

## 2. Materials and Methods

### 2.1. hDPSCs Isolation and Culture

Intact, decay-free third molars extracted from human participants aged 18–25 years were preserved in serum-free minimal essential medium (MEM; HyClone, Logan, UT, USA) and split. Pulp tissue was minced into small pieces using sterile surgical scissors and digested with 3 mg/mL collagenase type I (Sigma–Aldrich, St. Louis, MO, USA) for 30 min. Tissue pieces were seeded onto 35-mm^2^ cell culture plates at 37°C, in a 95% O_2_/5% CO_2_ incubator.

### 2.2. Primary Culture and Trigeminal Neuron Pretreatment

Primary trigeminal ganglion neurons were collected from 2- to 7-day-old Sprague–Dawley rats. After exposing the base skull, the bilateral trigeminal ganglia were isolated aseptically. The tissues were washed twice in precooled phosphate-buffered saline (PBS) without calcium and magnesium ions, containing 100 IU/mL each of penicillin and streptomycin. Trigeminal ganglia were cut into small fragments using sterilized ophthalmic scissors and digested in collagenase type I for 30 min at 37°C in a 5% CO_2_ incubator. We transfer tissues to a 15-mL tube with 1 mL DNAse I mixed with 1 mL trypsin for further digestion. After resuspension in Neurobasal A medium containing 2% B27™, the cells were counted and cultured on coverslips coated with poly-l-ornithine and laminin, to which 10 *μ*M cytosine arabinoside was added to further reduce non-neuronal cell growth. All experiments were carried out 6–7 days after dissection when the cells reached complete maturation.

### 2.3. Flow Cytometry

Fluorescent-conjugated monoclonal antibodies, including CD45 APC, CD73 FITC, CD90, and PerCP (BD Biosciences, San Jose, CA, USA), and STRO-1 PE (Santa Cruz Biotechnology, Dallas, TX, USA), were used. Single-cell suspensions (5 × 10^5^) were incubated at 37°C for 45 min. After resuspension in PBS, the cells were analyzed using a flow cytometer (FACSCanto II, BD Biosciences) and FlowJo software.

### 2.4. Osteogenic and Adipogenic Differentiation

Next, hDPSCs (2.5 × 10^5^ cells/well) were seeded in six-well plates. The mineralization induction medium contained 10 mmol/L *β* glycerophosphate, 100 nmol/L dexamethasone, and 50 *μ*mol/L l-ascorbic acid phosphate [18]. Alkaline phosphatase and Alizarin Red S staining were performed. Images were captured using a microscope (Olympus, Tokyo, Japan).

For adipogenic differentiation, we use the hDPSCs Adipogenic Differentiation Medium Kit (HUXDP-90031, Cyagen, Suzhou, China). Cells were first cultured in induction medium (containing insulin, 3-isobutyl-1-methylxanthine, and dexamethasone) for 3 days, then maintenance medium was changed to continue the culture for 1 day, this step was repeated within 20 days, and then induction medium was cultured for 7 days until large lipid droplets appeared. Oil Red O staining was performed after 30 days [18].

### 2.5. Differentiation of Schwann-Like Cells Derived from Dental Pulp Stem Cells

Next, hDPSCs at passages 2–3 were cultured in MEM without fetal bovine serum (FBS), containing 1 mM *β*-mercaptoethanol (*β*-ME; Sigma) for 24 hr. Subsequently, the cells were incubated in MEM with 10% FBS supplemented with 35 ng/mL all-trans retinoic acid (RA; Sigma) for 3 days. Next, cells were transferred to *α*-MEM containing 10% FBS with 5 *μ*M forskolin (Sigma), 10 ng/mL basic fibroblast growth factor (b-FGF; PeproTech, Rocky Hill, NJ, USA), 5 ng/mL platelet-derived growth factor AA (PDGFaa; MedChemExpress, NJ, USA), and 200 ng/mL recombinant human neuregulin-1 (NRG-1, Genscript, Nanjing, China). Finally, the cells were cultured for 2 weeks with medium changes every 2–3 days. Hereafter, hDPSCs that differentiated into Schwann-like cells are referred to as Schwann-like cells derived from dental pulp stem cells (SC-DPSCs).

### 2.6. Quantitative Real-Time PCR

Total RNA was extracted using TRIzol reagent (Invitrogen, Carlsbad, CA, USA) per manufacturer's protocol. The first-strand complementary DNA was generated using the PrimeScript® RT reagent Kit (Takara, Dalian, China). Real-time quantitative reverse transcription polymerase chain reaction (PCR) was performed with SYBR-Green Real-Time PCR Master Mix Plus (Takara) in a 20 *μ*L reaction mixture using an ABI7500 sequence detection system (Applied Biosystems, Darmstadt, Germany). The primer sequences are listed in Table 1. The relative expression levels were calculated by the 2^−*ΔΔ*Ct^ method.

### 2.7. Immunofluorescence Staining

The cells were incubated in 24-well plates on glass slides, fixed with 4% paraformaldehyde for 15 min, permeabilized with 0.5% Triton X-100 for 20 min at room temperature, and blocked with 5% bovine serum albumin for 30 min, followed by incubation with primary antibodies against neurofilament 200 (NF-200, 1 : 400, Proteintech, Wuhan, Hubei, China), *β*-III tubulin (TUBB3,1 : 200, Proteintech), and microtubule-associated protein 2 (MAP2, 1 : 200, Proteintech) overnight at 4°C. After washing, the sections were incubated with Alexa Fluor 594-coupled antimouse or antirabbit immunoglobulin secondary antibodies for 1 hr at room temperature. Finally, the sections were mounted with 60% glycerol and visualized under a confocal laser scanning microscope (Leica STELLARIS 5, Wetzler, Germany).

### 2.8. Enzyme-Linked Immunosorbent Assays

Culture media collected from the SC-DPSCs were analyzed using enzyme-linked immunosorbent assays for human brain-derived neurotrophic factor (BDNF; SCA011Hu, Cloud-Clone Corp., Wuhan, China), human glial cell line-derived neurotrophic factor (GDNF; SEA043Hu), and human neutrophin-3 (NT-3; SEA106Hu). The working solution on the substrate was covered with a new plate sealer and protected from light. The chemiluminescence signal was measured at a wavelength of 450 nm using a microplate reader (FLUOstar OPTIMA; BMG Labtech, Ortenberg, Germany); standard curves were generated.

### 2.9. Collection of Conditioned Medium

The conditioned media is the mixture of the collected media from SC-DPSCs and fresh neurobasal A media in a 1 : 1 ratio. Fresh neurobasal A media is the routine medium for culturing trigeminal neurons. The method of obtaining collected media is described as follows: hDPSCs were seeded in standard hDPSCs culture medium. The differentiation medium is the medium in which hDPSCs are induced toward SC-DPSCs. The media were collected on days 6 and 8 during the induction of hDPSCs toward SC-DPSCs and stored at −80°C.

### 2.10. Cell Counting Kit-8 Assay for Trigeminal Neuron Proliferation

Isolated trigeminal neurons were incubated in 96-well plates with a conditioned medium. The mixture of the collected medium from SC-DPSCs and fresh neurobasal A medium was 1 : 1. The cells were then incubated for 24, 48, 72, or 96 hr, and 10 *μ*L of Cell Counting Kit-8 solution was added to each well. The absorbance of all cell cultures in a 96-well plate was measured at a test wavelength of 450 nm using a microplate reader (BMG Labtech).

### 2.11. RNA Extraction, Library Construction, and RNA-Seq

RNA was extracted from the samples using the TRIzol reagent (Invitrogen) according to manufacturer's instructions. RNA integrity was assessed using an RNA Nano 6000 Assay Kit on an Agilent Bioanalyzer 2100 system (Agilent Technologies, Santa Clara, CA, USA). High-quality RNA was sent to BGI Genomics Co., Ltd., (Shenzhen, China) for cDNA library construction and sequencing. After secondary quality control, clean reads were filtered using the SOAPnuke filtering software. Hierarchical indexing for spliced alignment of transcripts was used to compare the RNA-seq reads with the reference genome. Pearson correlation coefficients were calculated for all gene expressions, and these coefficients were reflected as heat maps.

### 2.12. Functional Group Analysis

The Phyper function in R software (R Foundation for Statistical Computing, Vienna, Austria) was used for enrichment analysis to calculate *P*-values, and the false discovery rate was applied to evaluate the significance of the *P*-value. Transcripts with *P*-values ≤ 0.05 and threshold values ≥2-fold change were specified as differentially expressed transcripts. Differentially expressed protein-coding mRNAs between groups were identified using a histogram. GO and KEGG pathway analyses were conducted using topGO (version 2.18.0) (http://www.bioconductor.org/packages/release/bioc/html/topGO.html) and KOBAS (kobas3.0), respectively, to evaluate the biological relevance and functional pathways of the shared significant genes from our microarray data. Differentially expressed mRNAs were annotated based on their gene product attributes. According to the GO annotation results, DEGs were functionally classified based on molecular functions, cellular components, and biological processes. Furthermore, the biological functions of the genes are better understood through an integrated analysis of KEGG pathways and gene annotations. A protein correlation network was established using PPIs (https://cn.string-db.org/).

### 2.13. Statistical Analysis

Data interpretation and statistical analyses were performed using SPSS software (version 22.0; SPSS, Inc., Chicago, IL, USA). Data from experiments performed in triplicate are expressed as means and standard deviations. Student's *t*-tests were used to compare groups. *P*-values < 0.05 were considered statistically significant.

## 3. Results

### 3.1. Characterization of Primary Cultured hDPSCs

**Figure 1:**
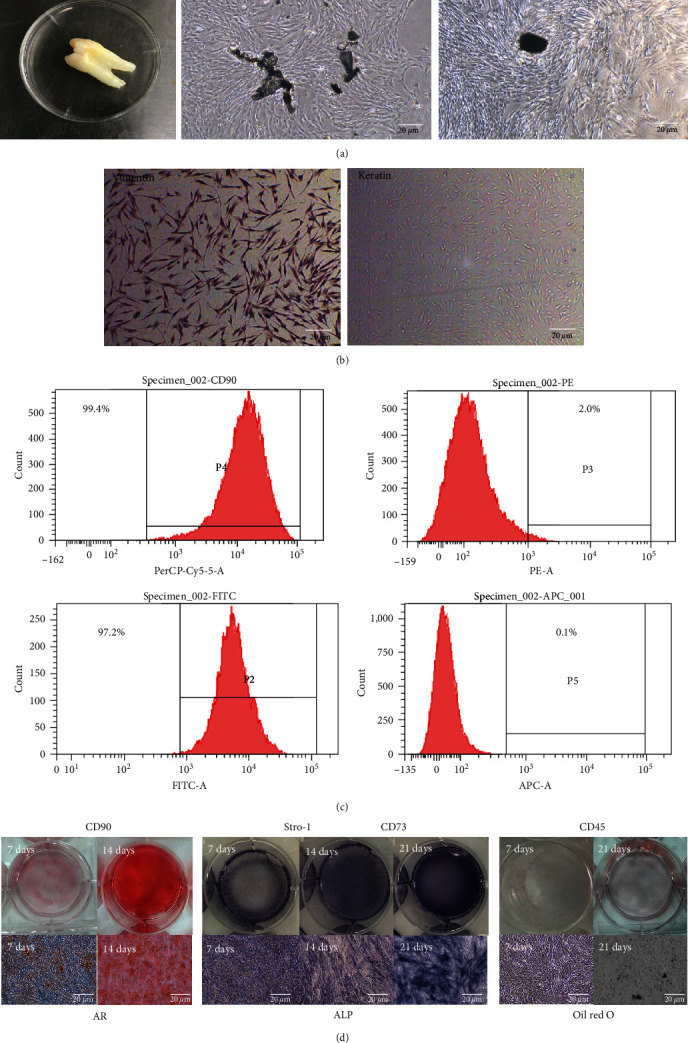
Culture and characterization of hDPSCs: (a) hDPSCs were isolated from the coronal pulp of human third molars via enzymatic digestion and tissue cultivation; (b) vimentin protein was positive in hDPSCs (immunohistochemistry staining); (c) CD73, CD90, STRO-1, and CD45 were analyzed by flow cytometry; and (d) hDPSCs were cultured in osteogenic or adipogenic induction medium. Scale bar: 20 *μ*m. hDPSC, human dental pulp stem cell.

Cells migrating from the edge of the tissue block were spindle-shaped and formed whirlpools (Figure 1(a)). Immunocytochemical staining revealed that the cells positively expressed vimentin and negatively expressed keratin (Figure 1(b)). HDPSCs were positive for CD73 (97.2%), CD90 (99.4%), while negative for CD45 (0.1%) and STRO-1 (2%), as assessed by flow cytometry (Figure 1(c)). Mineralized nodule formation and lipid-rich vacuole accumulation confirmed osteogenic and adipogenic hDPSC differentiation (Figure 1(d)).

### 3.2. SC Induction of hDPSCs

**Figure 2:**
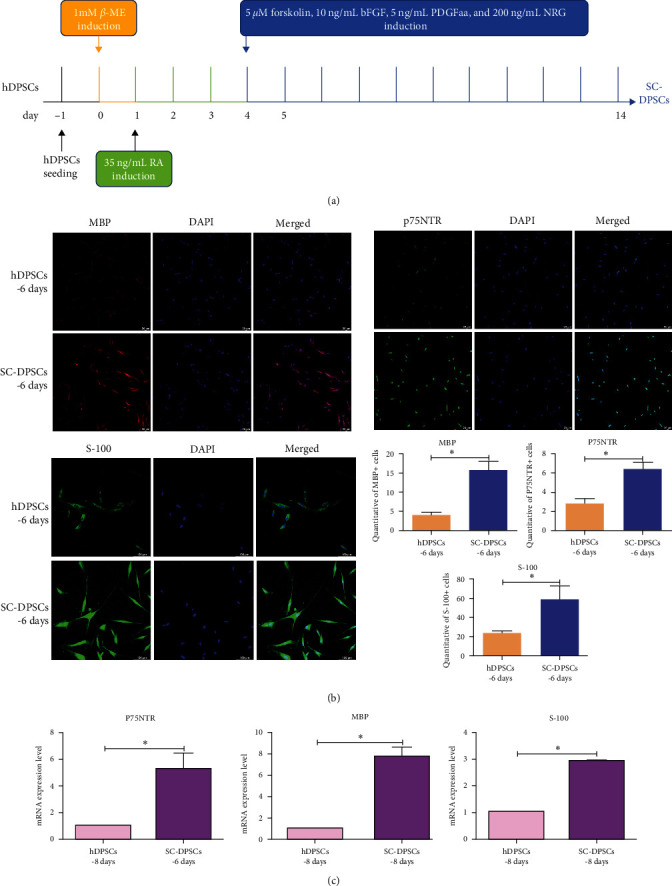
Differentiation of hDPSCs into glial cells: (a) schematic diagram of the culture assay. hDPSCs cultured in *β*-ME medium for ∼1 day before further seeding in RA culture medium for 3 days, followed by culture in Forskolin, b-FGF, PDGFaa, and NRG-1 medium. Cells and cell supernatant were collected for further characterization; (b) immunofluorescent staining was performed on hDPSCs and SC-DPSCs for the typical Schwann cell markers MBP, p75NTR, and S-100. Scale bar: 20 *μ*m. hDPSC, human dental pulp stem cell; PDGFaa, platelet-derived growth factor AA; SC, stem cell; (c) RT-PCR analysis of the expression of Schwann cell marker MBP, p75NTR, and S-100. b-FGF, basic fibroblast growth factor; PDGFaa, platelet-derived growth factor AA; NRG-1, neuregulin-1; MBP, myelin basic protein; p75NTR, p75 neurotrophin receptor; and *β*-ME, *β*-mercaptoethanol.  ^*∗*^*P* < 0.05.

Figure 2(a) shows a flowchart of the induction processes. *β*-ME was first added under serum-free conditions for 24 hr; RA was then added for 3 days, followed by PDGFaa. Immunofluorescent staining was performed using antibodies against myelin basic protein (MBP), p75 neurotrophin receptor (p75NTR), and S-100 (Figure 2(b)). MBP and S-100 expression increased in the cytoplasm and nucleus of the induced cells, and nuclear p75NTR levels increased significantly. The MBP, p75NTR, and S-100 mRNA expression levels increased on day 8 (Figure 2(c)). These results suggest that the induced hDPSCs secreted specific SC factors.

### 3.3. Nerve Growth Factor Secretion by SC-DPSCs

**Figure 3:**
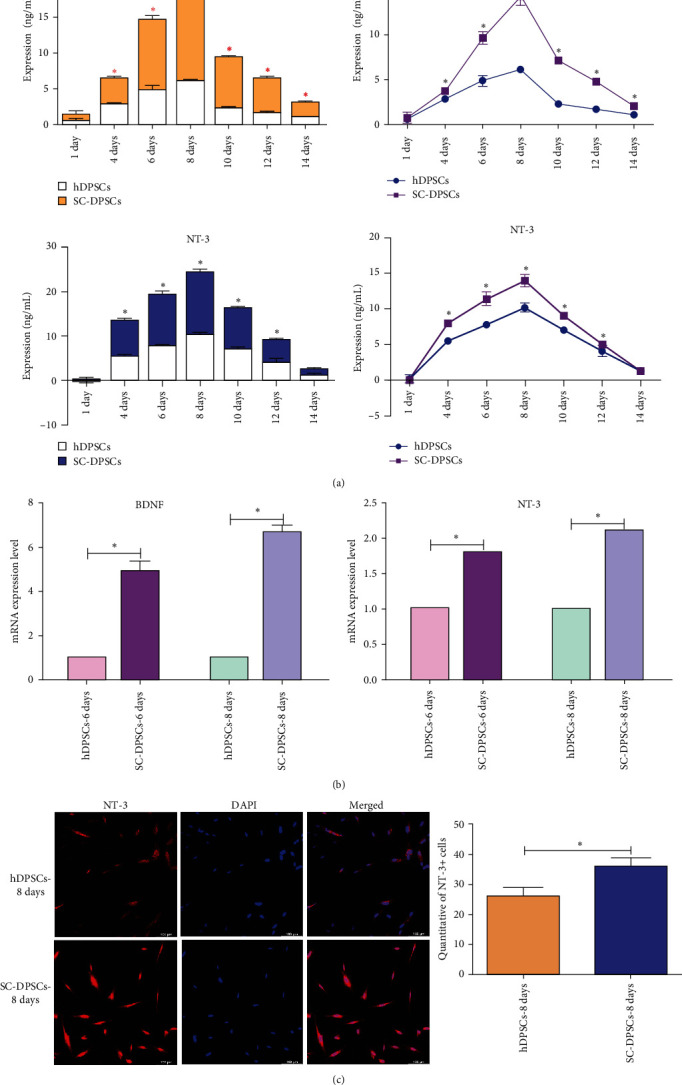
Neurotrophin expression in hDPSCs after induction: (a) enzyme-linked immunosorbent assays indicated a significant increase and dynamic tendency in BDNF and NT-3 levels after differentiation; (b) the expression of genes coding for BDNF and NT-3 in hDPSCs was analyzed by qRT-PCR; and (c) immunofluorescent staining was performed to detect NT-3 expression. hDPSC, human dental pulp stem cell; BDNF, brain-derived neurotrophic factor; and NT-3, neurotrophin-3.  ^*∗*^*P* < 0.05.

Compared with hDPSCs, BDNF, and NT-3 expression increased in SC-DPSCs from days 1–8 (Figure 3(a)). BDNF and NT-3 standard curves were generated (Figure S1(a)). During the induction period, BDNF and NT-3 expression increased (Figure 3(a)). These results were consistent with those of the real-time PCR (Figure 3(b)). Moreover, mRNA BDNF and NT-3 levels were elevated on days 6 and 8. Immunofluorescence analysis revealed that NT-3 expression increased on day 8 (Figure 3(c)). On days 2 and 4, there is no significant change in the expression of GDNF, but on days 6 and 8, there is a significant decrease in the expression of GDNF (Figure S1(b)). These results suggest that SC-DPSCs are superior to DPSCs regarding nerve growth factor secretion.

### 3.4. Neuronal Markers in Primary Rat Trigeminal Neurons

**Figure 4:**
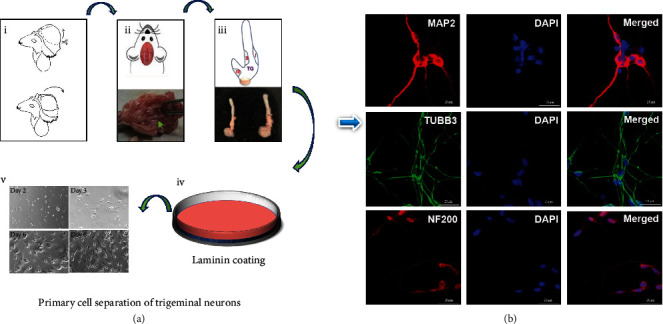
Isolation and identification of trigeminal nerve: (a-i) the skull was cut along the left and right sides, exposing the area between the eyes, and then the bone was lifted. Subsequently, the brain was removed. (a-ii) The base of the skull is exposed. (a-iii) This panel illustrates the structure and composition of the trigeminal nerve. (a-iv) Ornithine encapsulation was performed overnight, followed by the addition of a laminin coating. (a-v) The images depict trigeminal neurons at different culture stages. (b) Immunofluorescent staining reveals MAP2, TUBB3, and NF200 expression in trigeminal axons. Scale bar: 20 *μ*m. NF-200, neurofilament 200; TUBB3, *β*-III tubulin; and MAP2, microtubule-associated protein 2.

Figure 4(a) shows that the trigeminal neurons became larger and interwoven into a network covering the field of vision. On day 7, the neurons fully matured, expressing the specific markers for sensory neurons. MAP2 was mainly distributed in the axons and cytoplasm, whereas TUBB3 and NF-200 were primarily stained in the axons (Figure 4(b)). Cytosine arabinoside was used to remove the glial cells intermingled with the trigeminal nerve. Glial fibrillary acidic protein is a glial cell marker used to distinguish trigeminal neurons (Figure S2).

### 3.5. Indirect Coculture of Trigeminal Neurons and SC-DPSCs

**Figure 5:**
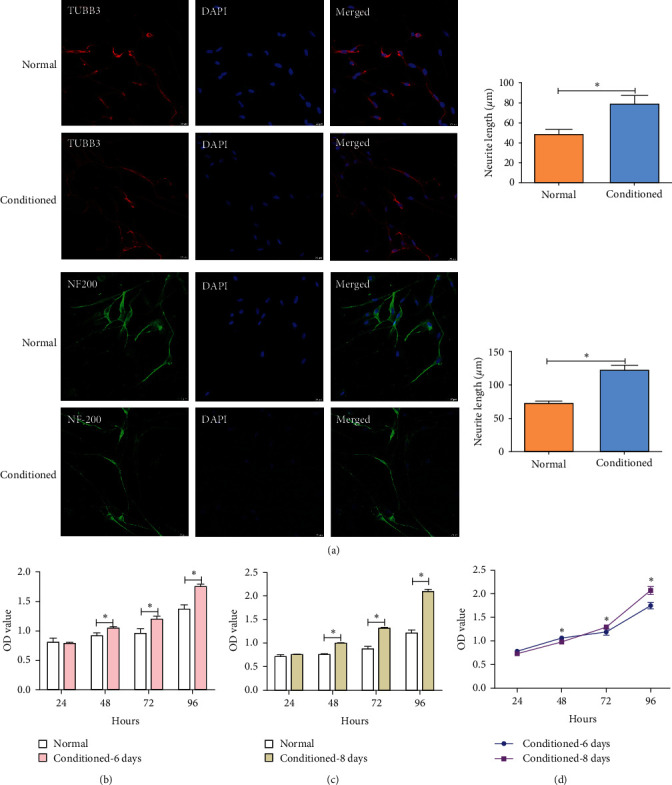
Trigeminal neuron growth after adding conditioned medium: (a) trigeminal axon growth (TUBB3, red color; NF200, green color; DAPI, blue color) is shown by immunofluorescent staining in the presence of cell supernatant of SC-DPSCs; (b) CCK-8 was used to analyze the trigeminal neuron growth on different days after adding a conditioned medium of 6 days; (c) CCK-8 was used to analyze the trigeminal neuron growth on different days after adding a conditioned medium of 8 days; (d) comparison of trigeminal neuron growth after addition of 6 days induction medium and 8 days induction medium. Scale bar: 20 *μ*m; SC-DPSCs, Schwann-like cells derived from dental pulp stem cells; NF-200, neurofilament 200; TUBB3, *β*-III tubulin; MAP2, microtubule-associated protein 2.  ^*∗*^*P* < 0.05.

Supernatants from SC-DPSCs and DPSCs were added to trigeminal neurons after the axon growth of the trigeminal neurons stabilized. After a 7-day period, axon elongation in trigeminal neurons was more prominent when the supernatant of SC-DPSCs was introduced (day 6), than when the supernatant of DPSCs was introduced (day 6; Figure 5(a)). Introduction of the supernatant of SC-DPSCs on day 6 promoted trigeminal neuronal growth at 48, 72, and 96 hr (Figure 5(b)). The axonal growth of the trigeminal nerve was longer after adding the SC-DPSC supernatant (Figure S3). The supernatant of SC-DPSCs on day 8 promoted trigeminal neuronal growth at 48, 72, and 96 hr, which was most prominent at 96 hr (Figure 5(b)). Introduction of the supernatant of SC-DPSCs on day 8 exhibited higher proliferative activity in trigeminal neurons than that on day 6 after 96 hr of incubation (Figure 5(b)–5(d)). SC-DPSCs exerted a more pronounced stimulatory effect on trigeminal neurons, with a longer interaction time with trigeminal neurons.

### 3.6. Signature Gene Network of SC-DPSCs and DPSCs

**Figure 6:**
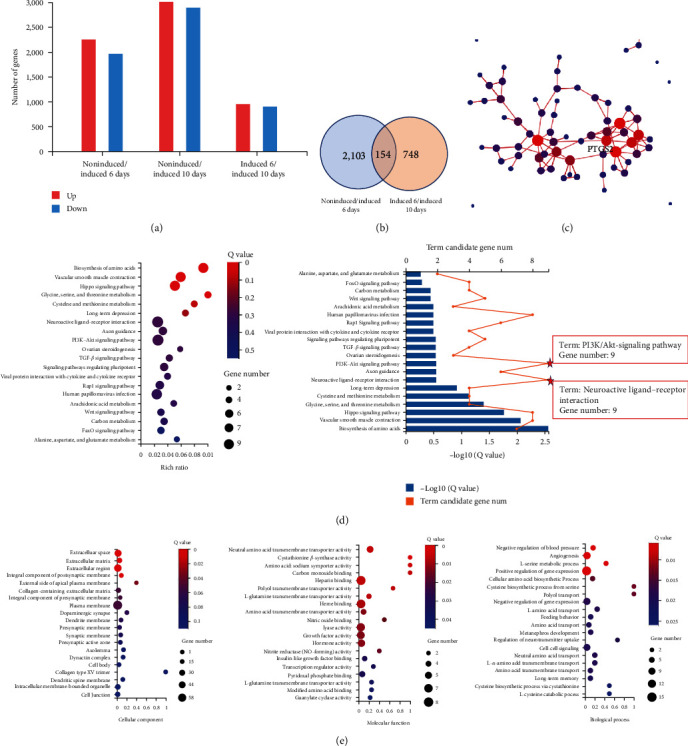
Candidate mRNA selection from RNA-seq analysis: (a) bar graph shows the gene expression in the 6-day and 10-day induced and noninduced groups; (b) the Venn diagrams illustrate 2,257 genes that were significantly upregulated between induced 6 days and noninduced conditions; 902 genes were downregulated between days 6 and 10 of induction (log2FC > 2; Q value < 0.001); (c) STRING database and cytoscape show the interaction of 154 genes, including one key gene; (d) Kyoto encyclopedia of genes and genomes pathway analysis of associated mRNAs of 154 genes (bubble chart and bar chart on the left and right sides, respectively); and (e) gene ontology enrichment analysis of associated mRNAs of 154 key genes (from left to right—cellular component, molecular function, and biological process). Induced 6 days means supernatant on the sixth day of differentiation of Schwann-like cells derived from dental pulp stem cells (SC-DPSCs); induced 10 days means supernatant on the tenth day of differentiation of SC-DPSCs.

We refined the mRNA candidates from the RNA-seq DEGs of 6 and 10 days induced and noninduced hDPSCs (Figure 6(a)). The sample correlation heat map showed little variation within each group; the box plot showed a roughly consistent dispersion of the data (Figures S4(a) and S4(b)). Based on the analysis of the RNA transcriptomes between days 6 and 10 of induction, 2,257 upregulated and 902 downregulated DEGs were identified. The Venn diagrams revealed 154 DEGs among these mRNAs (Figure 6(b)), among which, one had the most extensive interaction with the other genes and was located at the core of the PPI network (Figure 6(c)). The top enriched KEGG pathways consisted of nine genes associated with neuroactive ligand–receptor interactions and nine genes associated with the PI3K–Akt signaling pathway (Figure 6(d)). GO analysis revealed that the upregulated mRNAs enriched the cellular components, including the extracellular space and plasma membrane. This biological process is associated with the negative regulation of blood pressure, angiogenesis, and L-serine metabolic processes. The associated molecular functions included cystathionine *β*-synthase activity, amino acid sodium symporter activity, and carbon monoxide binding (Figure 6(e)). These RNA-seg results indicate that prostaglandin-endoperoxide synthase 2 (PTGS2) might be a critical gene in hDPSCs induced by SC-DPSCs. Neuroactive ligand–receptor interactions and complement and coagulation cascades may be the key pathways.

## 4. Discussion

The natural course of axonal repair and regeneration includes nerve end retraction, hemorrhage, inflammation, neurotoxic cytokine release, and fibrosis. During this process, the nerve cell body begins to regrow axons from the proximal stump to the distal end. SCs play critical roles in eliminating degenerated axons, myelin debris, and inhibitory factors at the distal end [14]. A recent in vivo study showed that incorporating SCs into engineered constructs can guarantee the functionality of nerve guidance conduits to promote axon regeneration [19]. Therefore, the role of SCs in nerve injury reconstruction, including secondary nerve degeneration, axonal regeneration, and remyelination, should be overemphasized.

Nevertheless, using primary SCs in cell-based neural regeneration is hindered by limitations including slow proliferation of SCs and morbidity of the donor nerve tissue [20]. Dental-derived stem cells originating from the neural crest may have higher neurogenic differentiation potential than that of other MSCs [21]. When stem cell from apical papilla, DPSCs, and periodontal ligament stem cells were used to treat rat sciatic nerve gap defects, they provided a substitute for SCs, offering benefits for peripheral nerve repair [22].

The hDPSCs originating from migrating neural crest cells are predisposed to differentiation into peripheral glial cells under the appropriate conditions [23]. Moreover, the high proliferative capacity, plasticity, multipotency, and immunoregulatory capabilities of hDPSCs make them excellent candidates for regenerative medicine, particularly in neural tissue engineering [24]. Martens et al. [25] reported the successful differentiation of hDPSCs into a SC-like phenotype [23]. In vitro, hDPSCs were differentiated into Schwann-like cells by adding various factors including *β*-ME, RA, growth media containing mixed growth factors, NRG, PDGFaa, forskolin, and b-FGF [25]. Woodbury et al. [26] first used *β*-ME and RA to successfully induce human and rat bone marrow MSCs to enter immature neurons in vitro. Here, hDPSCs were induced using *β*-ME and RA. When cells are treated with forskolin, cyclic adenosine monophosphate levels increase, thereby increasing mitotic gene expression, which was verified by sequencing results. The combination of FGF, NRG, forskolin, and PDGFaa synergistically promoted hDPSC differentiation into cells with SC-like characteristics.

The expression of multiple markers was evaluated to confirm that differentiated hDPSCs had a SC-like phenotype. MBP is synthesized and secreted by oligodendrocytes and is a marker of mature oligodendrocytes [27]. MBP is also synthesized and secreted by SCs and is present in the myelin sheath of peripheral nerves [28]. S-100 is an acidic calcium-binding protein secreted mainly by astrocytes in the central nervous system and SCs in the peripheral nervous system [29]. p75NTR is a low-affinity neurotrophic receptor expressed in glial and neuronal cells that regulates neuronal cell proliferation and differentiation and is associated with synapses and nerve formation via different signaling pathways [30]. Here, compared to undifferentiated hDPSCs, SC-DPSCs stained strongly positive for glial markers including MBP, p75NTR, and S-100, resembling SCs, demonstrating the successful morphological differentiation of hDPSCs into Schwann-like cells.

Dental pulp tissue is a source of neurotrophic factors that promote neuronal survival and neurite growth both in vitro and in vivo [31] and expresses nerve growth factors including NGF, BDNF, GDNF, and SDF-1 [32]. These factors are also associated with the axonal guidance of the trigeminal nerve, which enables perceptual facial function and motor control of the mandibular occlusal muscles [33]. Similarly, we observed BDNF and NT-3 secretion by undifferentiated hDPSCs. We found that SC-DPSCs had better neuroprotective and neurotrophic effects than that of undifferentiated DPSCs. Indeed, using a conditioned medium derived from SC-DPSC cultures significantly increased trigeminal cell survival more than that in undifferentiated DPSCs cultures and increased the axon length of the neurons.

Here, we used high-throughput sequencing to generate a signature profile of numerous mRNAs in SC-DPSCs and noninduced hDPSCs on days 6 and 10. We uncovered 2,257 mRNAs upregulated after 6 days in SC-DPSCs and noninduced hDPSCs. Based on logFC ≥ 2 and *P* ≥ 0.001, a total of 154 upregulated and downregulated genes were identified, and the correlations between the transcripts in the groups were examined. Among these, the key gene, PTGS2, was highly expressed.

PTGS2 encodes cyclooxygenase-2 and is extremely polymorphic, with a class of single-nucleotide polymorphisms in its regulatory regions [34]. PTGS2 is a key upstream enzyme in the prostaglandin synthesis pathway that converts arachidonic acid to prostaglandin H2, which is further converted by downstream enzymes to other prostaglandin such as prostaglandin D2 and prostaglandin E2 [35]. The inducible prostaglandin synthase PTGS2 mRNA transcript level was upregulated in mouse skin wounds up to 5 days later [35]. Wei et al. [36] identified a subpopulation of SC-like cells that highly expressed genes associated with SC proliferation, migration, and nerve regeneration, including PTGS2, PITX1, VEGFA, and FGF2, using single-cell RNA sequencing.

PTGS2 mRNA was among our top 20 enriched GO biological processes, implying its profound effect with great potential to induce hDPSCs differentiation into SCs. GO analysis revealed that the increased mRNAs were enriched for extracellular space, extracellular region, extracellular matrix, and external side of the apical plasma membrane (GO: cellular component); negative regulation of blood pressure, angiogenesis, and L-serine metabolic process (GO: biological process); and cystathionine *β*-synthase activity, amino acid: sodium symporter activity, and carbon monoxide binding (GO: molecular function). The most enriched KEGG pathways were neuroactive ligand–receptor interaction, PI3K/Akt-signaling pathway, Rap1 signaling pathway, axon guidance, and transforming growth factor-*β* signaling pathway.

We successfully differentiated DPSCs from Schwann-like cells both morphologically and functionally. SC-DPSCs express characteristic SC markers and promote trigeminal neurite outgrowth in vitro, providing translational potential for cell therapy for dental pulp nerve regeneration.

In summary, after differentiation into SC-DPSCs, hDPSCs secreted more nerve growth factors, BDNF, and NT-3 than before, significantly promoting trigeminal nerve axon growth. The key gene was PTGS2.

## Figures and Tables

**Table 1:** Sequence of various primers and oligonucleotides used in this study.

Name	Sequence (5′−3′)
*β*-actin F	CTCCCTGGAGAAGAGCTACGAGC
*β*-actin R	CCAGGAAGGAAGGCTGGAAGAG
BDNF F	GGC TTG ACA TCA TTG GCT GAC
BDNF R	CAT TGG GCC GAA CTT TCT GGT
NT-3 F	AAC GCG ATG TAA GGA AGC CA
NT-3 R	AGT GCTCGG ACG TAG GTT TG
P75NTR F	CCTACGGCTACTACCAGGATG
P75NTR R	CACACGGTGTTCTGCTTGT
S-100 F	TGGCCCTCATCGACGTTTTC
S-100 R	ATGTTCAAAGAACTCGTGGC
MBP F	GGCCGGACCCAAGATGAAAA
MBP R	CCCCAGCTAAATCTGCTCAGG

BDNF, brain-derived neurotrophic factor; NT-3, Neurotrophins-3; P75NTR, P75 neurotrophin receptor; MBP, myelin basic protein; F, forward; and R, reverse.

## Data Availability

The data used to support the findings of this study are available from the corresponding author upon request. The transcriptomic data from this study was deposited in NCBI Sequence Read Archive under accession SRA: PRJNA1079518.

## Supplementary Materials

Supplementary Materials Supplementary materials for culturing cells and detecting nerve growth factor.
