# Supplementary material for: The Effect of Differentiation of Human Dental Pulp Stem Cells to Glial Cells on the Sensory Nerves of the Dental Pulp
Source: Int J Dent. 2024 Apr 5;2024:3746794. doi: 10.1155/2024/3746794 (PMC11074855; doi:10.1155/2024/3746794)

Fig. S1. The expression of nerve growth factor was detected by ELISA. (A) A standard curve of BDNF and NT-3 secretion detected by ELISA after induction of hDPSCs into SCs. (B) the expression of GDNF secreted by hDPSCs after induction into SCs.


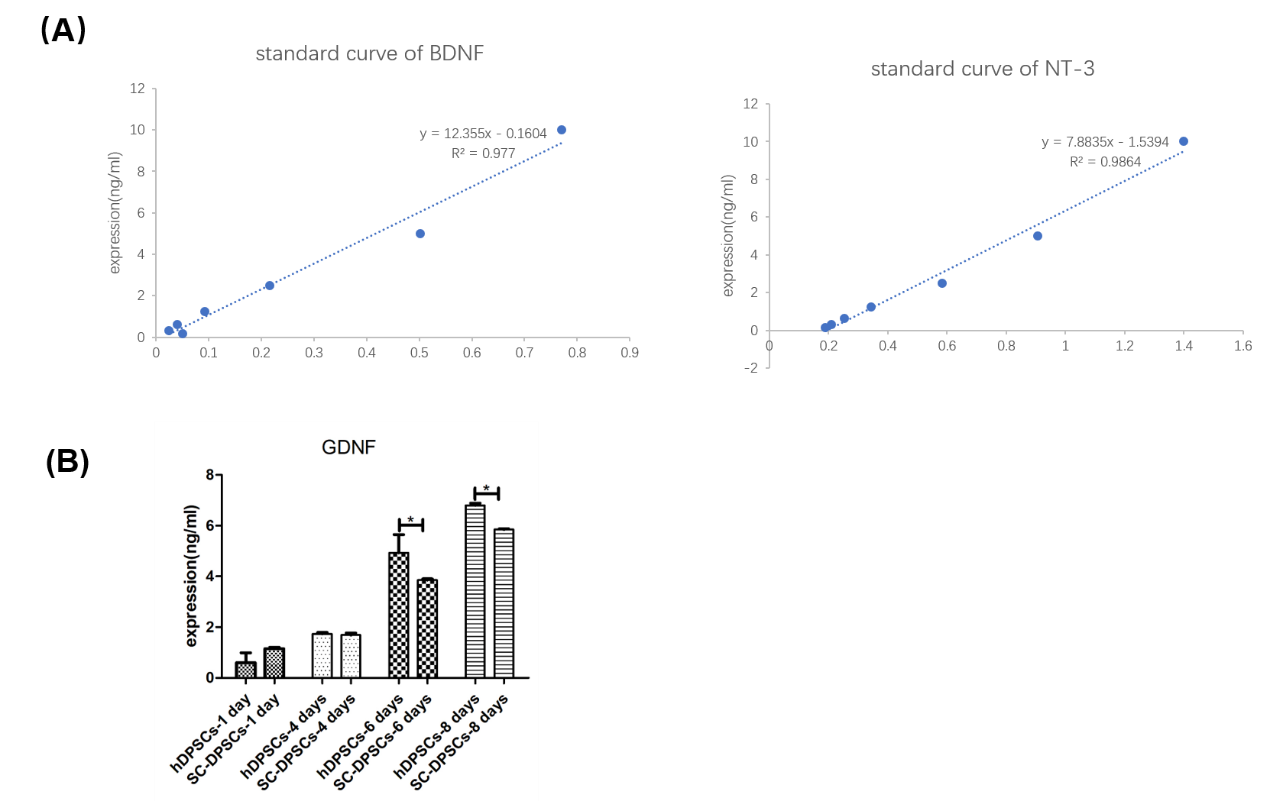


Fig. S2. Trigeminal nerves were harvested from neonatal SD rats for primary culture. Before cytarabine screening, the expression of glial cell marker GFAP was detected by immunofluorescence staining.


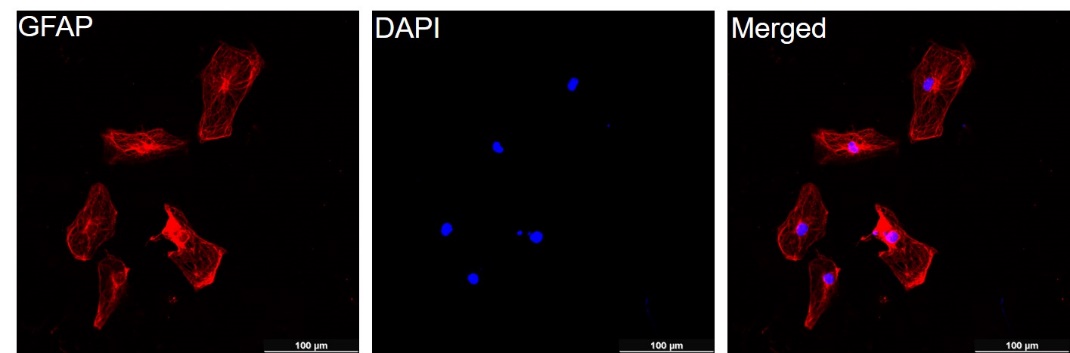


Fig. S3. After 6 days of induction, the supernatant of induced hDPSCs was mixed with trigeminal neuron medium in a 1:1 ratio to form the conditioned medium for trigeminal neuron culture. The results showed that the axons of trigeminal neurons grew significantly after the addition of the conditioned medium, and the extension length was longer than that of the control.


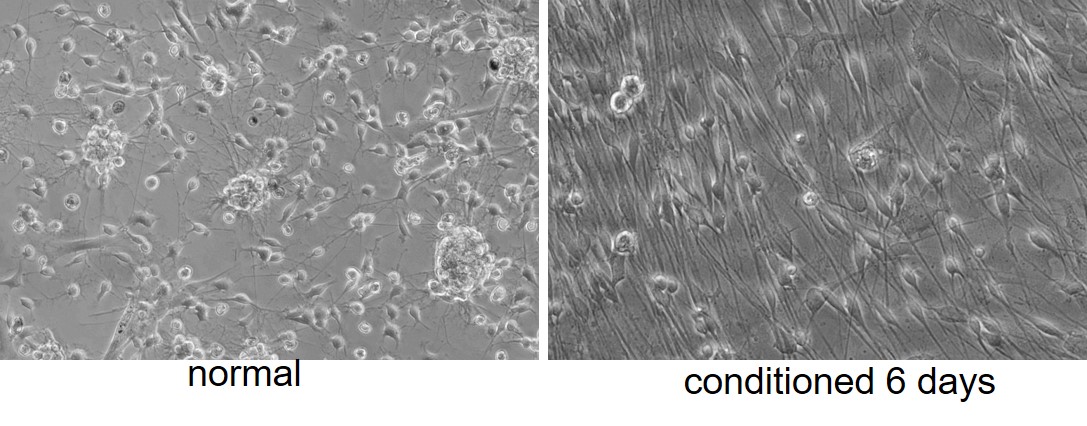


Fig. S4. Correlation heat map and box chart reflect the correlation and expression distribution of samples. (A) In order to reflect the correlation of gene expression between samples, Pearson correlation coefficients of all gene expression levels between each two samples were calculated, and these coefficients were reflected in the form of heat map. The correlation coefficient can reflect the similarity of overall gene expression among samples. The higher the correlation coefficient, the more similar the gene expression level is. (B) The boxplot shows the distribution of gene expression levels in each sample, and the degree of dispersion of data distribution can be observed.


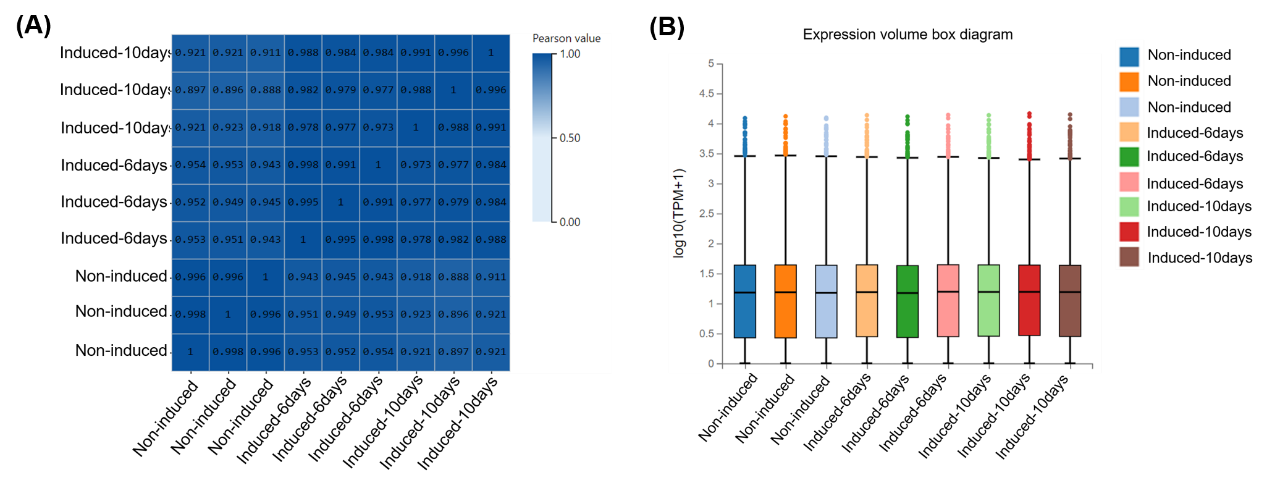

Supplement: Supplementary Materials — for culturing cells and detecting nerve growth factor. [file 3746794.f1.docx]
